# Aminoglycoside Resistance Among Clinical Bacterial Isolates in Sétif, Algeria: Epidemiology, Multidrug Resistance, and Virulence Features

**DOI:** 10.3390/antibiotics15020222

**Published:** 2026-02-17

**Authors:** Anfal Kara, Chiara Massaro, Naouel Boussoualim, Giovanni M. Giammanco, Rosa Alduina, Zineb Daoudi, Noussaiba Douadi, Fatma Gridi, Mohammad Raish, Byong-Hun Jeon, Hyun-Jo Ahn, Yacine Benguerba

**Affiliations:** 1 Laboratory of Applied Biochemistry, Faculty of Nature and Life Sciences, Ferhat Abbas University of Setif 1, Setif 19000, Algeria; karaanfel98@gmail.com (A.K.); naouel_24@yahoo.fr (N.B.); zdaoudi907@gmail.com (Z.D.); douadinoussaiba7@gmail.com (N.D.); fatmagrid026@gmail.com (F.G.); 2 Department of Biological, Chemical and Pharmaceutical Sciences and Technologies (STEBICEF), University of Palermo, Viale delle Scienze, Bldg. 16, 90128 Palermo, Italy; chiara.massaro01@unipa.it (C.M.); valeria.alduina@unipa.it (R.A.); 3 Department of Health Promotion, Mother and Child Care, Internal Medicine and Medical Specialties “G. D’Alessandro”, University of Palermo, 90127 Palermo, Italy; giovanni.giammanco@unipa.it; 4 National Biodiversity Future Center (NBFC), Piazza Marina 61, 90133 Palermo, Italy; 5 Department of Pharmaceutics, College of Pharmacy, King Saud University, P.O. Box 2457, Riyadh 11451, Saudi Arabia; mraish@ksu.edu.sa; 6 Department of Earth Resources and Environmental Engineering, Hanyang University, 222 Wangsimni-ro, Seongdong-gu, Seoul 04763, Republic of Korea; hjahn93@hanyang.ac.kr; 7 Laboratory of Biopharmaceutics and Pharmaceutical Technology (LBPT), Ferhat Abbas University of Setif 1, Setif 19000, Algeria

**Keywords:** antibiotic resistance, aminoglycosides, virulence factors, epidemiologic profile

## Abstract

**Background**. Antibiotic resistance is a growing global health challenge, complicating the management of infections. Aminoglycosides are increasingly associated with resistance, raising the risk of clinical complications and mortality in severe infections. This study aimed to characterize the epidemiological profile of 135 aminoglycoside-resistant clinical strains collected in Setif between 2021 and 2023. **Methods**. Antibiotic susceptibility testing was performed according to EUCAST guidelines, and phenotypic assays were conducted to assess key virulence traits, including biofilm formation and enzyme production. **Results.** Aminoglycoside resistance was more frequently observed in female patients (55.6%) and was found to be predominant among adults (68.1%). Urinary tract infections represented the main clinical presentation (76.3%), with *Escherichia coli* being the most common isolate (40.7%). High resistance rates were detected for amoxicillin (83%), amoxicillin–clavulanic acid (80%), cephalexin (74.8%), cefixime (71.1%), trimethoprim–sulfamethoxazole (74.8%), and gentamicin (72.6%). Conversely, chloramphenicol (53.3%), imipenem (47.4%), amikacin (47.4%), and piperacillin–tazobactam (31.1%) remained comparatively more effective. Multidrug resistance involving seven antibiotics occurred in 25.6% of isolates, with notable cross-resistance patterns between gentamicin and β-lactam antibiotics (5 out of 22). Genotypic analysis showed that 43% of isolates carried at least one β-lactamase gene, whereas 9.6% harbored a *qnr* determinant. Regarding virulence factors, isolates with low biofilm-forming ability were found to be the most common (62.96%). **Conclusion**. In conclusion, this study revealed substantial variations in aminoglycoside resistance in Setif, shaped by demographic, clinical, and bacteriological factors.

## 1. Introduction

Since their discovery, antibiotics have represented a pivotal advance in modern medicine, profoundly transforming the management of infectious diseases worldwide and significantly reducing both morbidity and mortality [1,2]. However, this medical progress is currently seriously threatened by the rapid emergence of antibiotic resistance, which has become a major global health crisis. This phenomenon is driven primarily by the overconsumption and misuse of antimicrobial agents [3].

The World Health Organization (WHO) has identified antimicrobial resistance among the ten main global health risks [4]. In 2019, antimicrobial resistance (AMR) was responsible for an estimated 700,000 deaths per year. It is assumed that, by 2050, this figure could rise to 20 million deaths, with an economic burden exceeding USD 2.9 trillion [5].

Among the groups of antibiotics particularly affected by this crisis are aminoglycosides. These broad-spectrum antibiotics are widely used in the treatment of severe infections [6], particularly those caused by Gram-negative bacteria [7]. They act by specifically binding to the 16S rRNA of the 30S ribosomal subunits, thereby disturbing protein synthesis [8]. Aminoglycosides are also recognized for their bactericidal activity and their synergistic interactions with β-lactams [9].

This study investigates the prevalence of aminoglycoside resistant bacteria among clinical isolates from Setif, Algeria, using an integrated phenotypic and genotypic approach. In addition to susceptibility testing, we performed multiplex PCR to document clinically relevant co resistance determinants, focusing on β lactamase genes and plasmid-mediated quinolone resistance markers (*qnr*).

## 2. Results

### 2.1. Distribution of Samples According to the Origin

**Figure 1:**
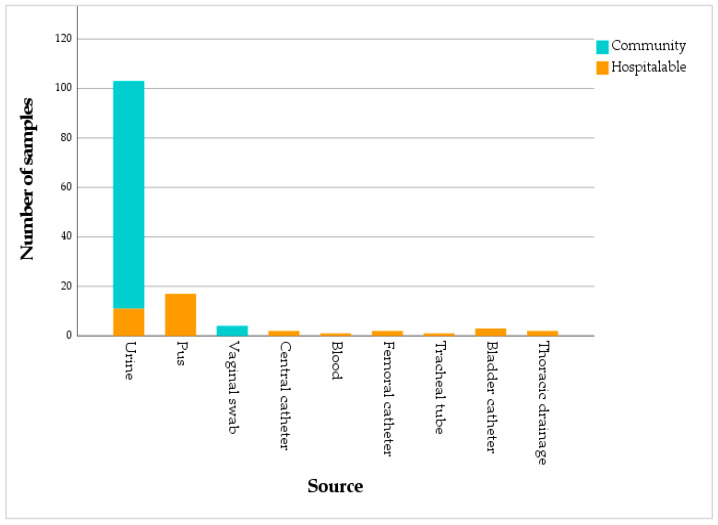
Distribution of samples by source and type of infection.

Most of the isolated samples originated from community-acquired infections (71.1%), while hospital-acquired infections (patients hospitalized for more than 48 h) accounted for 28.9%. Among community-associated cases, the vast majority of samples were urinary (95.8%), with vaginal specimens representing only 4.2%. In the hospital setting, samples were mainly pus (43.6%) and urine (28.2%), followed by bladder catheter specimens (7.7%). Samples linked to medical devices (femoral and central venous catheters, thoracic drainage) accounted for 5.1%, while blood and tracheal aspirates represented 2.6%. The association between infection type and sample source was statistically significant (*p* < 0.001, Chi-square test) (Figure 1).

### 2.2. Distribution and Prevalence of Bacterial Strains

**Figure 2:**
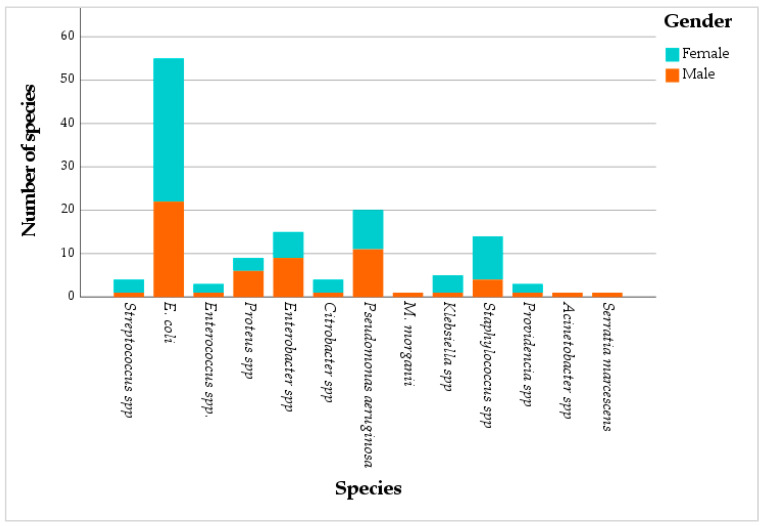
Distribution of bacterial strains by gender.

The majority of the samples were obtained from female patients, representing 55.6% of the study population, compared to 44.4% male patients. The isolates were predominantly recovered from adults (68.1%, *p* = 0.001, Chi-square test), followed by elderly people (20.7%) and children (11.1%). Patient ages ranged from 1 to 96 years, with a mean of 46.2 years. The mean ages within each subgroup were: 5.76 years for children, 40.88 years for adults, and 75.57 years for elderly patients. Urine specimens constituted the majority of all samples collected, accounting for 76.3% (Figure 2).

Out of 135 samples, 55.6% came from female patients and 44.4% came from male patients. *E. coli* is the most common pathogen in both groups, with (24.4%) in females and (16.3%) in males. Next are *Staphylococcus* spp. (7.4%) and *P. aeruginosa* (6.7%) in females, compared to *P. aeruginosa* (8.2%) and *Enterobacter* spp. (6. 7%) in males (Figure 2). Community-acquired infections were more frequent in females (39.3%) than in males (16.3%), while hospital-acquired infections represented (31.9%) in females and (12.6%) in males, respectively (Figure 2).

**Figure 3:**
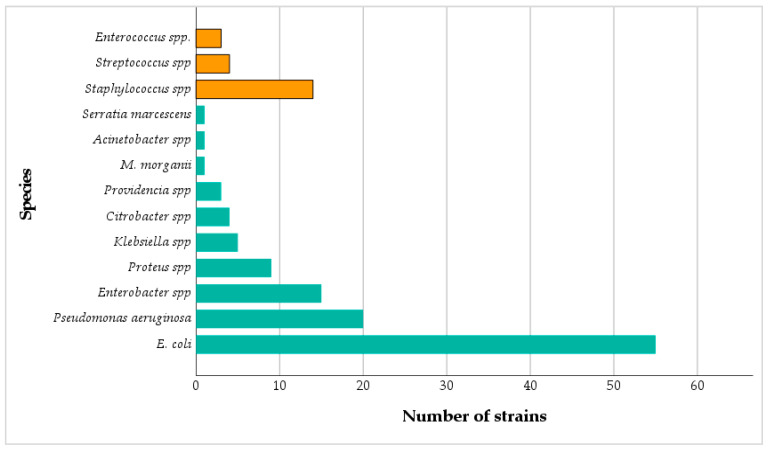
Distribution and prevalence of bacterial strains: Blue: Gram-negative strains, Orange: Gram-positive strains.

The analysis of the isolated bacterial species revealed a clear predominance of Gram-negative bacteria, which accounted for 114 isolates (84.4%) out of 135, while Gram-positive bacteria represented 21 isolates (15.6%). Among the identified species, *E. coli* was the most frequent (40.7%), followed by *P. aeruginosa* (14.8%), *Enterobacter* spp. (11.1%), and *Staphylococcus* spp. (10.4%). Less common isolates included: *Proteus* spp. (6.7%), *Klebsiella* spp. (3.7%), *Citrobacter* spp. and *Streptococcus* spp. (3.0% each). Additionally, *Providencia* spp. and *Enterococcus* spp. were each detected in 2.2% of cases, while *Serratia marcescens*, *Acinetobacter* spp., and *Morganella morganii* each accounted for only 0.7%. This distribution confirms a significant association between bacterial species and Gram classification (*p* < 0.001, Chi square test) (Figure 3).

### 2.3. Antibiotic Resistance Profile

**Figure 4:**
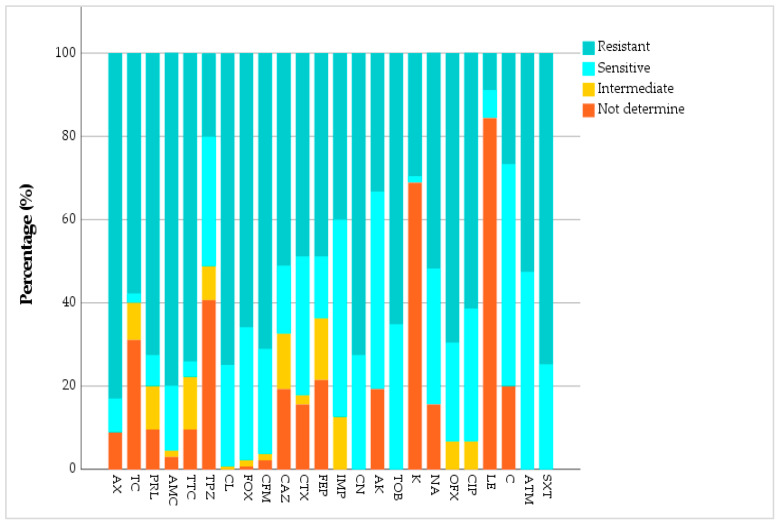
Distribution of sensitivity profiles of the 135 bacterial strains tested against different antibiotics (AX: amoxicillin, TC: ticarcillin, PRL: piperacillin, AMC: amoxicillin–clavulanic acid, TTC: ticarcillin–clavulanic acid, TPZ: piperacillin+ tazobactam, CL: cefalexin, FOX: cefoxitin, CFM: cefixime, CAZ: ceftazidime, CTX: cefotaxime, FEP: cefepime, IMP: imipenem, CN: gentamicin, TOB: tobramycin, AK: amikacin, K: kanamycin, NA: nalidixic acid, OFX: ofloxacin, CIP: ciprofloxacin, LE: levofloxacin, C: chloramphenicol, ATM: aztreonam, SXT: trimethoprim–sulfamethoxazole).

Our study focused on 135 bacterial strains resistant to at least one of the four tested aminoglycosides to determine their susceptibility to other classes of antibiotics, in order to detect multidrug resistance. Among the aminoglycosides, gentamicin (CN) showed the highest resistance rate at 72.6%, followed by tobramycin (TOB) at 65.2%, and kanamycin (K) at 29.6%. Amikacin (AK) was the most effective, with a resistance rate of 33.3% and the highest sensitivity at 47.4% (Figure 4).

High rates of resistance were also observed among penicillins. Of the 135 strains analyzed, 83% (*p* ≤ 0.01) were resistant to amoxicillin (AMX), and 80% (*p* ≤ 0.01, Chi-square test) to amoxicillin–clavulanic acid (AMC). Piperacillin (PRL) and ticarcillin–clavulanic acid (TTC) exhibited resistance rates of 72.6% (*p* ≤ 0.01, Chi square test) and 74.1% (*p* ≤ 0.01, Chi square test), respectively. In contrast, piperacillin–tazobactam (TPZ) showed only 20% of strains resistant and 31.1% (*p* ≤ 0.01, Chi square test) sensitive.

Analysis of susceptibility to cephalosporins revealed a high proportion of resistant strains among the 135 isolates analyzed. A resistance rate of 74.8% (*p* ≤ 0.01, Chi-square test) was observed for cephalexin (CN), 71.1% (*p* ≤ 0.01, Chi square test) for cefixime (CFM), and 65.9% (*p* ≤ 0.01, Chi square test) for cefoxitin (FOX). The lowest resistance rates were observed for cefotaxime (CTX) and cefepime (FEP), both at 48.9% (Figure 4).

Sensitivity to quinolones varied depending on the specific antibiotic tested. Among the 135 strains analyzed, 69.6% (*p* ≤ 0.01, Chi-square test) showed resistance to ofloxacin (OF), 61.5% (*p* ≤ 0.01, Chi-square test) to ciprofloxacin (CIP), and 51.9% (*p* ≤ 0.01, Chi-square test) to nalidixic acid (NA). In contrast, levofloxacin (LE) showed the lowest resistance rate, with only 8.9% (*p* ≤ 0.01, Chi-square test) of resistant strains (Figure 4).

High resistance was also observed for trimethoprim–sulfamethoxazole (SXT), with 74.8% of resistant strains (*p*< 0.001, Chi-square test). Imipenem (IMP) showed a resistance rate of 40% (*p* < 0.001, Chi-square test), while 52.6% of isolates were resistant to aztreonam (ATM). Chloramphenicol (C) showed the lowest resistance rate, with only 26.7% of strains resistant (*p* < 0.001, Chi-square test).

### 2.4. Analysis of Multi-Resistance Profile

In this study, each strain was tested against 24 antibiotics from different classes, allowing a comprehensive assessment of their resistance profiles. All isolates (100%) exhibited multidrug resistance (MDR), defined as resistance to at least one antibiotic in three or more different classes. A consistent number of isolates (25.9%; *p* < 0.001, Chi-square test) were resistant to seven different antibiotics, followed by those resistant to six (17.8%) and eight antibiotics (16.3%). Resistance to a larger number of antibiotics (nine or ten) was less common, observed in 6.7% and 0.7% of strains, respectively. Conversely, resistance to a smaller number of antibiotics, such as three (5.2%) or four (14.1%), was observed in fewer isolates (Table 1).

### 2.5. Correlations Between Aminoglycosides and Other Classes of Antibiotics

The correlations between four aminoglycosides: gentamicin (CN), kanamycin (K), amikacin (AK), and tobramycin (TOB), and antibiotics from different classes: penicillins, cephalosporins, carbapenems, quinolones, polymyxins, and fosfomycin. Significant correlations (*p* < 0.05, Pearson test) were indicated. Among the penicillins, ticarcillin shows a correlation with gentamicin (TC–CN, n = 52; *p* = 0.025, Pearson test). Cephalosporins showed strong associations between cephalexin and gentamicin (CL–CN, n = 81; *p* < 0.001, Pearson test), cefixime and gentamicin (CFM–CN, n = 77; *p* = 0.002, Pearson test), as well as cefoxitin and gentamicin (CX–CN, n = 73; *p* = 0.001, Pearson test). Another significant association is observed between cefotaxime and tobramycin (CTX–TOB, n = 42; *p* = 0.045, Pearson test) (Table 2).

For carbapenems, imipenem is correlated with gentamicin (IMP–CN, n = 35; *p* = 0.011) and with tobramycin (IMP–TOB, n = 39; *p* = 0.008, Pearson test). Regarding quinolones, levofloxacin showed a weak association with gentamicin (LE–CN, n = 12; *p* = 0.042, Pearson test), while ciprofloxacin presents a moderate correlation with tobramycin (CIP–TOB, n = 62; *p* = 0.023, Pearson test) (Table 2).

This indicates that some bacterial strains were resistant to both aminoglycoside and other antibiotic classes. In particular, the highest number of significant co-occurrences of resistance to other antibiotics (5 out of 22) was observed among gentamicin-resistant strains, including several β-lactams (penicillins and cephalosporins) and quinolones (Table 2).

### 2.6. Molecular Detection of Resistance Genes

Out of 135 strains, (58/135, 43%) have at least one β-lactamase gene, while (13/135; 9.6%) occurred at least one *qnr* gene. The aminoglycoside-resistant *E. coli* isolates, β-lactamase and quinolone resistance genotypes showed significant variation (*p* < 0.001, Chi-square test), most strains lacked β-lactamase genes (57%), while 18.5% carried a single gene, 11.1% had two, 8.1% three, 3% four, and 2.2% five genes. In contrast, the majority (90.4%, *p* ˂ 0.001, Chi-square test) lacked quinolone resistance determinants, with only 6.7% and 3% carrying single and double gene combinations, respectively (Table 3).

Among the aminoglycoside-resistant isolates, β-lactamase genes were frequently detected, with *blaTEM* (35.6%) being the most prevalent (*p* = 0.0001, Chi-square test), followed by *blaCTX-M II* (11.9%), *blaCMY-II* AmpC-type (11.1%), *blaSHV* (9.6%) and *blaOXA* (7.4%). In contrast, quinolone resistance genes were less common, with *qnrB* (8.1%) predominating, while *qnrD* (3.7%) and *qnrS* (0.7%) were infrequent, and *qnrA* and *qnrC* were not detected (Table 4).

Out of 58 strains that harbored at least 1 β-lactamase gene, 56.9% (33/58) present the β-lactamase genotype by gene combination. A variety of β-lactamase gene combinations were identified among aminoglycoside-resistant isolates. Double-gene associations were most frequent, particularly *blaTEM*/*blaSHV* (15.2%) and *blaTEM*/*blaCTX-M I* (9.1%). The predominant triple combination, *blaTEM*/*blaCMY II*/*blaCTX-M II* (15.2%), revealed the co-expression of ESBL and AmpC-type β-lactamases. More complex profiles, including quadruple (12.1%) and quintuple (9.1%) gene combinations, such as *blaTEM*/*blaOXA*/*blaSHV*/*blaCMY II*/*blaCTX-M II*, were also detected (Table 5). Among the *qnr*-positive strains, 30.8% (4/13) harbored *qnrB/qnrD* combination, which was the only *qnr* combination.

The distribution of β-lactamase genes among aminoglycoside-resistant and aminoglycoside-susceptible isolates revealed marked differences, among gentamicin-resistant isolates, a significant association (*p* < 0.05, Chi-square test) was observed with several genes, including *blaTEM* (34 isolates), *blaOXA* (8 isolates), *blaSHV* (12 isolates), *blaCTX-M I* (7 isolates), and *blaCTX-M II* (12 isolates). For tobramycin-resistant isolates, a similar pattern was observed, with significant associations between resistance and the presence of *blaTEM* (32 isolates), *blaOXA* (9 isolates), *blaCMY II* (13 isolates), and *blaCTX-M II* (14 isolates) (*p* < 0.05, Chi square test).

In contrast, amikacin-resistant isolates did not show statistically significant associations with any of the tested genes, although *blaTEM* (24 isolates) and *blaCTX-M IV* (3 isolates) were relatively frequent in resistant strains compared with susceptible. Kanamycin-resistant isolates were rare and exhibited limited gene carriage, mainly *blaTEM* (6 isolates) and *blaSHV* (3 isolates) (Table 6).

### 2.7. Biofilm Production

Out of a total of 135 samples, and among the Gram-positive strains, 12.59% produce biofilm, while 2.96% do not. In contrast, among the Gram-negative ones, 73.33% produce a biofilm, compared to 11.11% that do not. Most of the strains come from urine samples, with 103 cases (76.3%), of which 87 (84.5%) were biofilm producers and 16 (15.5%) were non-producers. Pus represented the second most frequent source with 17 isolates (12.6%), of which 16 (94.1%) were producers. The vaginal swabs accounted for 4 strains (3%), of which 3 (75%) were producers. Other less frequent sources, such as central catheters, femoral catheters, and thoracic drains (2/2 each), as well as tracheal tubes (1/1) and blood isolation (1/1), only presented biofilm-producing strains (100%). Vesical catheters (2/3) also showed a majority of producing strains.

The evaluation of the biofilm production capacity of the 135 strains shows that the majority of the isolates were weak producers (62.96%), followed by moderate producers (17.04%), non-producers (14.07%), and strong producers, which were rarer (5.93%). The species *E. coli*, the most represented (40.7%), mainly has low production (72.7%). A similar trend is observed in *P. aeruginosa* and *Enterobacter* spp., with 50% and 73.3% of low-producing strains, respectively. Conversely, *Staphylococcus* spp. stood out with a relatively high proportion of high-producing strains (21.4%).

### 2.8. Enzymatic Activity

The analysis of enzymatic activities showed an uneven distribution according to the type of enzyme. The production of hemolysin was observed in more than half of the strains (54.1%), including (28.9%) α-hemolysin and (25.2%) β-hemolysin, while (45.9%) of the strains showed no hemolytic activity. In contrast, the production of protease did not show a significant difference, with similar proportions between producer strains (49.6%) and non-producer strains (50.4%). The production of lecithinase was much less frequent, found in only (24.4%) of the strains (*p* < 0.001, Chi square test). Similarly, a minority of strains express lipolytic activity (10.4%) compared to a majority of non-producing strains (89.6%) (*p* < 0.001, Chi-square test) (Table 7).

## 3. Discussion

The data showed that the majority of isolated bacterial strains (71.1%) come from infections contracted in the community. This reflects the high prevalence of community-acquired infections, particularly urinary infections, which account for almost all (95.8%) of the analyses in this group. The predominance of urinary infections is consistent with the trends observed at the national level, where *E. coli* is often responsible for these infections, especially in young or elderly females. This trend is confirmed by a recent Tunisian pediatric study, which showed a majority of female cases among patients with *E. coli* urinary infections, with a high frequency in young children and marked resistance to common antibiotics [10].

On the other hand, hospital-acquired infections (28.9%) are much less frequent than community-acquired infections. The analyses mainly come from pus (43.6%), which suggests frequent wound or surgical site infections in the hospital setting. Nosocomial urinary infections (28.2%) remain common as well, often linked to the use of urinary catheters, a well-known risk factor. A Moroccan study showed that urinary catheterization is responsible for 43% of nosocomial infections in Africa. The identified risk factors include advanced age, poor hygiene, medical history (such as diabetes or past urinary infections), as well as the lack of training of healthcare personnel [11].

The presence of analyses from invasive medical devices (such as femoral, central catheters, or thoracic drains) indicates a significant problem in the hospital setting. Infections related to medical devices represent a significant portion of healthcare-associated infections, particularly in intensive care units, where they are feared for their severity and the high risk of sepsis [12]. Infections associated with invasive medical devices, such as venous catheters, urinary catheters, and mechanical ventilation systems, are primarily caused by the formation of bacterial biofilms. These biofilms, which form on the devices, confer increased resistance to antimicrobial treatments to the bacteria [13].

The results of this study showed that most of the samples were taken from female patients; these results align with those of other studies, where, out of 214 recorded cases, 99 (46.3%) were males and 115 (53.7%) were females [14]. This trend may also be related to the fact that females seek healthcare more often than males and are three times more likely to have reported undergoing a health check-up [15]. A higher prevalence of *E. coli* urinary infections was observed in females (66.33%, n = 52) compared to males (30.67%, n = 23), across all age groups, by Jamil et al. (2018), confirming the observation of a higher proportion of samples from females in this study [16].

In terms of age distribution, adults (18–64 years) represented 68.1% of bacterial isolates, followed by the elderly and children. This distribution is significant (*p* = 0.001), confirming that middle-aged adults are the population most affected by bacterial infections. These results are consistent with those of Zhan et al. (2024), who found an increased prevalence among individuals in the younger age groups, particularly in the 50–69 age range, showing the highest proportion (57.3%) [17]. Moreover, 76.3% of the samples were urinary, indicating a high frequency of urinary infections in this population, especially among adults, as also reported by Jamil et al. (2018), who observed a high prevalence of *E. coli* urinary infections in females [16], and Zhan et al. (2024), who highlighted an increased frequency of these infections in middle-aged individuals [17].

The higher frequency of infections in females may be due to anatomical and physiological factors, such as the shortness of the urethra, its proximity to the anus, and changes during pregnancy, which increase the risk of urinary infections [14], and the predominance of *E. coli* in females is explained by the colonization of the area around the urethra by bacteria of intestinal origin, with *E. coli* being a normal component of the intestinal flora. The anatomical characteristics of females favor the ascent of these bacteria to the bladder [18].

*Enterobacteriaceae* represented 68.8% of the isolated bacteria in our study. A study conducted at the military hospital in Meknes showed that Enterobacteriaceae constitute approximately 68% of positive urine isolates in urinary tract infections [19]. Overall, Gram-negative bacilli were predominant (84.4%), particularly *E. coli* (40.7%), the main cause of urinary infections which is consistent with the results presented by Almutawif and Eid (2023), where Gram-negative bacteria were also the most frequently isolated group (76.14%), while Gram-positive bacteria accounted for 22.7% of the cases [20]. The predominance of Enterobacterales family especially *E. coli* bacteria due to its proximity to the urethra and its ability to adhere to the cells of the urinary epithelium, thus facilitating its migration to the bladder [21]. Other bacilli such as *Pseudomonas* spp. and *Enterobacter* spp. were also common, especially in hospital settings, where they often exhibit antibiotic resistance [22]. Gram-positive cocci, which are less numerous (15.6%), were mainly represented by *Staphylococcus* spp., often involved in infections related to medical devices [23].

The antibiogram results showed a high prevalence of bacterial resistance to the tested antibiotics, particularly to amoxicillin with 83% of resistant strains, confirming a concerning trend already observed. In the study by Kara et al. (2024) [24]. Piperacillin also showed high resistance, reaching 73.25% in the same study, which confirms the 72.6% obtained in our results [24]. Furthermore, in a study conducted in Mali by Goita et al. (2025) [25], *E. coli* strains exhibited a resistance rate of 57.14% to ticarcillin–clavulanic acid, which is concerning, but is slightly lower than the 89.54% observed for ticarcillin alone [25]. Our results showed limited resistance to piperacillin–tazobactam (20%), making it one of the most effective antibiotics against the tested strains. This observation aligns with that of Kara et al. (2024) [24], who showed that the combination of tazobactam with piperacillin significantly reduced bacterial resistance from 73% to 19%; tazobactam inactivates the bacterial β-lactamase enzymes, allowing piperacillin to remain effective against otherwise resistant bacteria [26].

The evaluation of the sensitivity of the 135 isolated strains to cephalosporins revealed very high resistance rates. These results are in agreement with those reported by Angho et al. (2024) [27], where more than 80% of the *Enterobacteriaceae* isolates exhibited resistance to cephalosporins. This high resistance is mainly due to the production of extended-spectrum β-lactamases (ESBL), detected in 68.3% of the strains isolated in this study, which showed a marked alteration in the effectiveness of these antibiotics [27]. On the other hand, lower resistance rates were observed for cefotaxime and cefepime (48.9%), these data correspond to those of a study conducted in Nigeria on *P. aeruginosa*, where the resistance rate to cefepimes was 28% [28].

The results showed varying levels of resistance depending on the tested fluoroquinolones. Ofloxacin, ciprofloxacin, and nalidixic acid, which showed resistance rates of 69.6%, 61.5%, and 51.9%, respectively. These results were consistent with those of Deku et al. (2022) in Ghana, who reported a resistance of 51.1% to ciprofloxacin, 51% to nalidixic acid in *E. coli* isolated from various infections [29]. Similarly, in the Ethiopian study by Hailemariam et al. (2021), resistance to ciprofloxacin reached 63.6% in *E. coli* and 80.9% in *K. pneumoniae* [30].

Regarding imipenem, a 40% rate of resistant strains has been noted. This data should be interpreted with caution, as it varies according to bacterial species and geographical contexts. In *E. coli*, Kara et al. (2024) [24] specifically studied 86 strains already resistant to imipenem. For *P. aeruginosa*, variable rates have been reported: Ugwuanyi et al. (2021) [28] noted imipenem resistance in 25.6% of strains isolated in Nigeria, while Vaez et al. (2015) observed a higher resistance, reaching 55.6% of hospital isolates in Iran [31].

The analysis of the 135 bacterial strains showed a worrying prevalence of antibiotic multi-resistance profile, with all strains (100%) being resistant to at least one antibiotic from three different families, these results correspond to a recent meta-analysis conducted in West Africa, which estimated the overall prevalence of multidrug-resistant bacteria at 59%, with significant variations between countries and sample types [32]. A significant portion of the bacterial strains studied exhibited resistance to six–eight antibiotics, reflecting a worrying trend towards multidrug resistance. These data confirm the global trends reported by WHO which highlight an increase in multidrug-resistant infections in healthcare facilities [33]. A recent review in West Africa also confirms this trend [34]. In the study by Ahmed et al. (2023) on 89 *K. pneumoniae*, 57.3% exhibit an MDR phenotype during COVID-19 [35]. According to Saeli et al. (2024), the prevalence of aminoglycoside-resistant *P. aeruginosa* isolates was 48%, of which 94.7% exhibited multidrug resistance (MDR) [36].

The analysis of correlations between aminoglycosides and other antibiotics showed significant cross-resistance, especially between gentamicin and several β-lactams such as cephalexin and cefixime. This co-resistance is often linked to common genetic mechanisms, such as ESBLs or enzymes modifying aminoglycosides [37]. Correlations between carbapenems (imipenem) and aminoglycosides also suggest an increase in multidrug-resistant strains, a phenomenon confirmed by a recent meta-analysis on infections caused by Gram-negative bacilli [38]. Finally, the observed links between quinolones and aminoglycosides indicate cross-resistance mechanisms such as efflux pumps, supported by recent research on the effect of cross-exposure to antibiotics [39].

The distribution of resistance determinants among the studied isolates revealed a significantly higher prevalence of β-lactamase genes compared with quinolone resistance determinants (*p* < 0.001 for both), with *blaTEM* being the most frequent (35.6%), consistent with reports from Switzerland [40] where aminoglycosides-resistant strains presented both ESBL and AmpC phenotypes, where *blaTEM* was the predominant β-lactams resistance machine followed by *blaCTX-M*. Strong association was found between the co-carriage of *blaOXA-1* β-lactamase type and *aac(60)-Ib-cr*, which compromises amikacin and tobramycin resistance [41].

Gram-negative bacteria dominated the analyzed samples, with a high proportion of strains capable of producing a biofilm. This observation is consistent with the data reported by Maione et al. (2023), which showed that among 1039 community samples, 96.2% of the isolates were Gram-negative compared to only 3.8% being Gram-positive [42]. Similarly, Dumaru et al. (2019) reported that, out of 314 *E. coli* isolates, 38% represented the most common strain [43].

Consistent to our results, Joshi et al. (2021) reported that 47.44% of the strains isolated from urine samples produced a biofilm [44]. Similarly, Belbase et al. (2017) observed that 46.1% of *S. aureus* isolates from pus or wound samples had this capability [45]. Most of our strains were weak biofilm producers, particularly *E. coli*, *P. aeruginosa*, and *Enterobacter* spp., while *Staphylococcus* spp. showed a higher proportion of strongly producing strains. These results were supported by several studies. Dumaru et al. (2019) showed that 62.73% of the tested isolates were positive for biofilm production, with a strong involvement of *Klebsiella* spp. and *Pseudomonas* spp. [43]. Karigoudar et al. (2019) also reported that, out of 100 urinary *E. coli* isolates, 69% were capable of producing a biofilm [46]. Macias-Valcayo et al. (2022) observed that 97% of Gram-positive studied strains formed biofilm [47], Murthy et al. (2024) reported 22% of biofilm producers in *S. aureus* strains [48]. Biofilms exhibit strong antibiotic resistance by limiting drug penetration, inactivating antibiotics, harboring dormant cells, and upregulating efflux pumps and resistance genes [49].

The production of extracellular enzymes is a key factor in bacterial pathogenicity. In this study, proteases were the most frequently produced (49.6%), a result consistent with those of Bertelloni et al. (2021) who reported (46.0%) [50]. Lecithinase activity was observed in 24.4% of the isolates, the same rate recorded for *Clostridium perfrengens* [51]. The production of lipases remained rarer (10.4%), although it can reach 52.0% according to Bertelloni et al. (2021), which reflects variability related to strains or conditions. Regarding hemolysis, Bertelloni et al. (2021) also indicate a dominance of the mixed α/β profile, followed by the β type and the α type [50]. Finally, Rahman et al. (2024) showed that lipase and protease-producing strains were more resistant to antibiotics, suggesting a link between enzymatic virulence and antibiotic resistance [52].

## 4. Materials and Methods

This study was conducted in several geographical locations around Setif province in the northeastern of Algeria; the collection was distributed on seven medical diagnostic laboratories, and three government hospitals. The study was approved by the Ethics and Deontology Committee in University Ferhat Abbas of Setif-1 under the study registered with the number of the paper, UFAS1/09/03/2023/ETH-Deon-A-301, and informed written consent was obtained from each participant.

### 4.1. Samples Collection and Bacteria Identification

During a three-year period from 2021 to 2023, 135 samples were selected for this study. All clinical samples: urine, pus, vaginal swab, catheters (central, femoral, bladder), blood, thoracic drainage, and tracheal tube, were collected under aseptic precautions. The samples were taken from patients of different ages and of both sexes. Patient information (age, sex, nature of sample, date of collection, patient type) was recorded in samples register at the microbiology laboratory, a positive culture without contamination respecting sterility criteria and showing decreasing sensitivity to one aminoglycoside agent were included in this study. The following samples were excluded from this study: samples with incomplete clinical or laboratory data; samples that did not meet the quality control or sterility criteria; samples which were not maintained at the required temperature or for the required duration of conservation before analysis; multiple samples from the same patient. All samples were cultured on non-selective and selective media such as nutrient agar, Hektoen agar, MacConkey agar, or Chapman agar and incubated at 37 °C for 24 h.

Identification of isolates was done by microscopic examination, colony morphology, Gram staining, motility and biochemical tests: indole production, mannitol, citrate utilization, glucose, sucrose, lactose fermentation in TSI agar, catalase, oxidase, urease and gas production. Then, the identified strains were preserved in a nutrient broth containing 30% sterile glycerol.

### 4.2. Antimicrobial Susceptibility Testing

Antimicrobial susceptibility tests were performed on Mueller–Hinton agar using the Kirby-Bauer disk diffusion method. A total of 24 antimicrobial agents were tested, including penicillins: amoxicillin (AX, 25 µg), ticarcillin (TC, 75 µg), and piperacillin (PRL, 100 µg). β-lactam/inhibitor combinations were tested: amoxicillin–clavulanic acid (AMC, 30 µg), ticarcillin–clavulanic acid (TTC, 85 µg), and piperacillin–tazobactam (TPZ, 110 µg). Cephalosporins were tested, including cefalexin (CL, 30 µg) as a first-generation agent, cefoxitin (FOX, 30 µg) as a second-generation agent, cefixime (CFM, 5 µg), ceftazidime (CAZ, 30 µg), and cefotaxime (CTX, 30 µg) as third-generation agents, and cefepime (FEP, 30 µg) as a fourth-generation agent. Carbapenems included imipenem (IMP, 10 µg); aminoglycosides included gentamicin (CN, 10 µg), amikacin (AK, 30 µg), tobramycin (TOB, 10 µg), and kanamycin (K, 30 µg); quinolones and fluoroquinolones included nalidixic acid (NA, 30 µg), ciprofloxacin (CIP, 5 µg), ofloxacin (OFX, 5 µg), and levofloxacin (LEV, 5 µg); phenicols included chloramphenicol (C, 30 µg), monobactam aztreonam (ATM, 30 µg), and the sulfonamide combination trimethoprim–sulfamethoxazole (SXT, 25 µg).

For this test, a bacterial inoculum was prepared from young culture for 20 ± 4 h in a non-selective culture medium and placed in a saline solution containing 0.9% sodium chloride. The surface of Mueller–Hinton agar plates (TM-Media, Delhi, India) was inoculated with the bacterial inoculum by swabbing within 15 min of the inoculum preparation. The swab was moved three times over the entire surface of the agar plate. The plate was then rotated 60° after each stroke to distribute the inoculum. Antibiotic disks were selected according to each species’ appropriate applicability; they were then placed on the agar surface. The antibiotics were selected according to the species’ appropriate applicability, with the plates applied using sterile forceps, pressing each disk down. The plates were then incubated at 35 ± 2 °C for 20 ± 4 h. Finally, after measuring the different inhibition zones with a ruler after the incubation period and comparing them with the critical diameter, the strain resistance data were interpreted according to the European Committee for Antimicrobial Susceptibility Testing (EUCAST) guidelines for 2022 [53]. They were categorized as follows: resistance (R), susceptible (S), or intermediate (I). This study included isolates with decreased sensitivity to at least one agent in an aminoglycoside family (resistant or intermediate phenotypes). Using the definition of multidrug-resistant bacteria (MDR) [8], our strains were classified as MDR strains.

### 4.3. Molecular Assays for Resistance Gene Screening by Multiplex PCR

Antimicrobial resistance genes (ARGs) were analyzed in isolates classified as resistant or intermediate to at least one antimicrobial agent (β-lactams and quinolones) by Kirby–Bauer testing. Genomic DNA was released from colonies using a heat–lysis method adapted from Woodman et al. (2016) [54] for each isolate. One–two colonies grown overnight on nutrient agar (Oxoid, Milan, Italy) were suspended in sterile water (100 µL), heated at 99 °C for 15 min, and centrifuged at 10,000× *g* for 10 min. The resulting supernatant was used as a DNA template. DNA integrity was checked by agarose gel electrophoresis, DNA samples showing a single, intact high-molecular-weight band without visible smearing were considered acceptable for further analyses. Concentration and purity were measured with a NanoDrop 2000c (Thermo Fisher Scientific, Waltham, MA, USA). Successful extraction was confirmed by PCR amplification of the 16S rRNA gene using primers F1 and R12 [55].

The presence of β-lactamase genes was assessed by two multiplex PCR assays: set 1 targeting *blaCTX-M IV*, *blaTEM*, *blaOXA*, and *blaSHV*; set 2 targeting *blaCMY II*, *blaCTX-M I*, *blaCTX-M II*, and *blaDHA*, as described by Kim et al. (2009) [56]. Plasmid-mediated quinolone resistance determinants (*qnrA*, *qnrB*, *qnrC*, *qnrD*, *qnrS*) were analyzed by singleplex PCR (Table 8). Each PCR was performed in 25 µL containing DreamTaq Buffer, dNTPs, primers (adjusted depending on singleplex or multiplex design), DreamTaq DNA Polymerase (Thermo Fisher Scientific), and 2 µL of template DNA. The amplification program included an initial denaturation, 35 cycles of denaturation, annealing at the primer-specific temperature and extension, followed by a final elongation step.

### 4.4. Biofilm Production

Biofilm formation is an important factor in the virulence of some bacteria and plays a significant role in increasing antibiotic resistance and host immune defense. To detect biofilm-forming capacity, a 96-well microtiter plate (TrustBio Corporation Ltd, Shanghai, China) was used, as described by Stepanović and et al. (2007) and Türkel et al. (2018) [58,59]. After growing bacterial strains in non-selective agar at 37 °C for 24 h, and after verifying the purity of the strains, a 0.5% McFarland solution in Brain Heart Infusion broth BHIB (Liofilchem, Abruzzo, Italy) supplemented with 2% glucose was prepared. A measure of 200 µL of each culture was then placed in microtiter plates with polystyrene wells (three wells for each bacterial isolate) and incubated at 37 °C for 24 h. Three wells were filled with sterile BHIB and designated as the negative control. All biofilm assays were performed in triplicate and repeated in at least three independent experiments to ensure reliability.

The medium was discarded after incubation, and the wells were washed three times with distilled water. To stabilize the adherent cells, 150 µL of methanol was added for 20 min. At room temperature, the biofilms were stained with a 0.2% (*w*/*v*) crystal violet solution for 15 min. The crystal violet was then removed, the wells were rinsed with sterile distilled water, and the plates were left to air-dry. The biofilms were then dissolved in 150 µL of 95% ethanol, and the absorbance was measured at 570 nm using an ELISA reader (BioTek, El Dorado Hills, CA, USA). The results were divided into four categories by comparing the measured OD with the negative control (ODc). Based on optical density (OD), four levels of biofilm production were distinguished. Strains with an OD equal to or lower than that of the ODc were classified as non-biofilm producers. Those with an OD higher than the ODc but less than twice (2 ODc) were considered as low biofilm producers. Moderate biofilm production was defined when the OD was between two and four times that of the ODc. Finally, strains with an OD greater than four times the control (4 ODc) were categorized as high-output biofilm producers.

### 4.5. Hemolysin Production

The tested isolates were cultured on blood human agar base (BHIB), which was prepared by adding 5% human blood to a blood agar base at a temperature of 45–50 °C. These isolates were incubated at 37 °C for 18–24 h. Hemolytic activity was determined by colony appearance and the presence of halos surrounding the colonies on blood agar [60,61]. Three phenotypes were observed: complete lysis of red blood cells, characterized by a clear zone around the colonies (β-hemolysis); partial lysis, forming a greenish halo due to the reduction of hemoglobin to methemoglobin (α-hemolysis); no visible change around the colonies, indicating the absence of lysis (γ-hemolysis).

### 4.6. Protease, Lecithinase and Lipase Production

The ability of the studied strains to produce proteases was determined on skimmed milk agar plates, which consist of peptone, yeast extract, and agar. After sterilization, 100 mL/L of ultra-high-temperature (UHT) sterilized milk was added to the base at a temperature ranging from 45 to 50 °C. The tested strains were then cultured and incubated at 37 °C for 24 h. The presence of a clear zone around the bacterial colonies indicates protease activity. *E. coli* ATCC 25922 was used as a negative control, while *P. aeruginosa* ATCC 27853 served as a positive control [62]. And to determine the lecithinase and lipase production capacity of the studied strains, they were cultured on egg yolk agar (EYA) plates (TM-Media, Delhi, India). A non-selective agar was used, to which egg yolk emulsion (egg yolk + sterile saline) was added at 45–50 °C. The cultures were then incubated at 37 °C for 24 h. The appearance of halos around the colonies indicates lecithinase activity. *Bacillus cereus* and *E. coli* ATCC 25922 were used as positive and negative control, respectively, while the appearance of an iridescent sheen on the surface of the colony indicates lipase activity. *P. aeruginosa* ATCC 27853 and *S. aureus* ATCC 29213 were used as the positive and negative control [63].

### 4.7. Statistical Analysis

Statistical analysis of resistance was performed using SPSS version 27 to calculate frequencies of qualitative variables. Pearson’s and chi-square tests were used when necessary to compare percentages in bivariate analysis. A *p*-value less than or equal to 0.05 was considered statistically significant.

## 5. Conclusions

This study analyzed the distribution and characteristics of bacteria resistant to aminoglycosides in the Setif region. The findings revealed notable variations in resistance rates. From a demographic perspective, females were more affected than males, with a clear predominance of resistance among adult patients. Clinically, urinary tract infections were the most frequently diagnosed, as urine samples represented the majority of the isolates. Bacteriological analysis indicated that Gram-negative bacteria, primarily *E. coli*, *P. aeruginosa*, and *Enterobacter* spp., were the most prevalent. In vitro phenotypic tests demonstrated a markedly high level of resistance to gentamicin, while amikacin showed the most effective antibacterial activity. Multidrug resistance involving seven antibiotics was the most commonly observed pattern, and gentamicin exhibited the strongest cross-resistance with other antibiotic classes, especially β-lactams. Regarding virulence factors, the majority of strains were characterized by low biofilm production, while enzymatic activities showed heterogeneous profiles.

This work provides an integrated picture of aminoglycoside resistance in the Setif region by combining epidemiological surveillance with phenotypic characterization, targeted genotypic screening of major co-resistance determinants. This combined strategy strengthens the interpretation of resistance patterns in a clinically relevant setting and supports future surveillance efforts by helping prioritize aminoglycoside resistance determinants for expanded molecular screening and mechanistic investigation.

## Figures and Tables

**Table 1:** Analysis of the distribution of isolates according to their level of multidrug resistance.

Antibiotic Classes with Resistance to at Least One Molecule	3	4	5	6	7	8	9	10
**n. isolates**	7	19	18	24	35	22	9	1
**Percentage (%)**	5.2	14.1	13.3	17.8	25.9	16.3	6.7	0.7
***p***	<0.001							

**Table 2:** Co-occurrence of aminoglycoside resistance with resistance to other antibiotic groups.

		Number			
Antibiotics		Aminoglycosides			
		CN	K	AK	TOB
**Penicillins**	**AMX**	79	23	34	71
	**AMC**	76	28	32	69
	**TTC**	71	23	30	71
	**PRL**	69	24	31	70
	**TC**	52 *	0	31	50
	**TPZ**	21	1	11	21
**Cephalosporins**	**CFM**	77 *	30	26	62
	**CX**	73	25	31	56
	**CAZ**	53	15	29	48
	**CTX**	54	12	19	42 *
	**FEP**	50	10	28	48
	**CL**	81 *	29	28	64
**Carbapenem**	**IMP**	35 *	8	25	39 *
**Quinolones**	**OF**	70	30	31	65
	**CIP**	63	26	25	62 *
	**NA**	52	19	23	47
	**LE**	12 *	9	6	10
**Phenicols**	**C**	29	4	8	21
**Sulfamide**	**SXT**	78	32	31	64
**Monobactam**	**ATM**	55	22	23	50

*: Statistically significant (*p* < 0.05); AX: amoxicillin; TC: ticarcillin; PRL: piperacillin; AMC: amoxicillin–clavulanic acid; TTC: ticarcillin + clavulanic acid; TPZ: piperacillin+ tazobactam; CL: cefalexin; FOX: cefoxitin; CFM: cefixime; CAZ: ceftazidime; CTX: cefotaxime; FEP: cefepime; IMP: imipenem; CN: gentamicin; TOB: tobramycin; AK: amikacin; K: kanamycin; NA: nalidixic acid; OFX: ofloxacin; CIP: ciprofloxacin; LE: levofloxacin; C: chloramphenicol; ATM: aztreonam; SXT: trimethoprim–sulfamethoxazole.

**Table 3:** Frequency of single and multiple β-lactamase (*blaTEM*, *blaSHV*, *blaOXA*, *blaCTX-M* groups *I*, *II* and *IV*, *blaCMY II*, *blaDHA*) or quinolone resistance gene carriage in aminoglycoside-resistant isolates.

β-Lactamase Gene			Quinolone Determinants		
Genotype	n (%)	*p*	Genotype	n (%)	*p*
**No gene**	77 (57)	˂0.001	**No gene**	122 (90.4)	˂0.001
**Single gene**	25 (18.5)		**Single gene**	9 (6.7)	
**Double gene**	15 (11.1)		**Double gene**	4 (3)	
**Triple gene**	11 (8.1)				
**Quadruple gene**	4 (3)				
**Quintuple gene**	3 (2.2)				

**Table 4:** Distribution of β-lactamase and quinolone resistance genes among aminoglycoside-resistant isolates.

Genotype	Genes	n (%)	*p*
**β-lactamase genes**	*blaCTX-M IV*	5 (3.7)	0.0001
	*blaTEM*	48 (35.6)	
	*blaOXA*	10 (7.4)	
	*blaSHV*	13 (9.6)	
	*blaCMY II*	15 (11.1)	
	*blaCTX-M I*	8 (5.9)	
	*blaCTX-M II*	16 (11.9)	
	*blaDHA*	4 (3)	
**Quinolone resistance gene**	*qnrA*	0 (0)	0.6
	*qnrB*	11 (8.1)	
	*qnrC*	0 (0)	
	*qnrD*	5 (3.7)	
	*qnrS*	1 (0.7)	

**Table 5:** Multiple β-lactamase gene associations in aminoglycoside-resistant isolates.

Gene Combination Type	Genotype Combination	n (%)
**Double genes**	*blaTEM/blaCTX-M I*	3 (9.1)
	*blaTEM/blaSHV*	5 (15.2)
	*blaSHV/blaCTX-M I*	1 (3.0)
	*blaTEM/blaOXA*	2 (6.1)
	*blaCTX-M IV/blaTEM*	2 (6.1)
	*blaTEM/blaCTX-M II*	1 (3.0)
	*blaCTX-M II/blaDHA*	1 (3.0)
**Triple genes**	*blaTEM/blaOXA/blaDHA*	1 (3.0)
	*blaTEM/blaCMY II/blaCTX-M II*	5 (15.2)
	*blaSHV/blaCMY II/blaCTX-M II*	1 (3.0)
	*blaCTX-M IV/blaTEM/blaOXA*	1 (3.0)
	*blaOXA/blaCMY II/blaCTX-M II*	2 (6.1)
	*blaTEM/blaSHV/blaCTX-M I*	1 (3.0)
**Quadruple genes**	*blaTEM/blaCMY II/blaCTX-M I/blaCTX-M II*	2 (6.1)
	*blaTEM/blaCMY II/blaCTX-M II/blaDHA*	1 (3.0)
	*blaTEM/blaOXA/blaCMY II/blaCTX-M II*	1 (3.0)
**Quintuple genes**	*blaTEM/blaOXA/blaSHV/blaCMY II/blaCTX-M I*	1 (3.0)
	*blaTEM/blaOXA/blaSHV/blaCMY II/blaCTX-M II*	2 (6.1)

**Table 6:** Comparative distribution of β-lactamase genotypes among aminoglycoside-resistant and aminoglycoside-susceptible isolates.

		P*-blaCTX-M IV*	P*-blaTEM*	P*-blaOXA*	P*-blaSHV*	P*-blaCMY II*	P*-blaCTX-M I*	P*-blaCTX-M II*	P*-blaDHA*
**GEN**	**R**	2	34 *	8 *	12 *	11	7 *	12 *	3
	**S**	3	13	2	1	4	1	4	1
**AK**	**R**	3	24	4	6	5	4	6	1
	**S**	2	22	6	7	9	3	9	3
**TOB**	**R**	3	32 *	9 *	8	13 *	6	14 *	2
	**S**	2	16	1	5	2	2	2	2
**K**	**R**	0	6	0	3	1	2	1	0
	**S**	0	0	0	0	0	0	0	0

P-*bla* gene: positive-*bla* gene strain, R: resistant, S: sensitive, *: statistically significant with *p* ˂ 0.05.

**Table 7:** Distribution of strains according to the production of extracellular enzymes.

Enzymes		n (%)	*p*
**Hemolysin**	Negative production	62 (45.9%)	0.007
	α-Hemolysin	39 (28.9%)	
	β-Hemolysin	34 (25.2%)	
**Protease**	Non-producers	68 (50.4%)	0.931
	Producers	67 (49.6%)	
**Lecithinase**	Non-producers	102 (75.6%)	<0.001
	Producers	33 (24.4%)	
**Lipase**	Non-producers	121 (89.6%)	<0.001
	Producers	14 (10.4%)	

**Table 8:** Primers and PCR conditions used for resistance *qnr* genes [57].

Gene	Sequence	T (°C)	Amplicon Size (bp)
***qnrA*_F**	ATTTCTCACGCCAGGATTTG	55	516
***qnrA*_R**	TGCCAGGCACAGATCTTGAC		
***qnrB*_F**	CGACCTKAGCGGCACTGAAT	55	515
***qnrB*_R**	GAGCAACGAYGCCTGGTAGYTG		
***qnrC*_F**	GGGTTGTACATTTATTGAATC	50	446
***qnrC*_R**	TCCACTTTACGAGGTTCT		
***qnrD*_F**	CGAGATCAATTTACGGGGAATA	50	581
***qnrD*_R**	AACAAGCTGAAGCGCCTG		
***qnrS*_F**	GACGTGCTAACTTGCGTGAT	56	118
***qnrS*_R**	TGGCATTGTTGGAAACTTG		

## Data Availability

All data generated or analyzed in this study are included in the published article, and additional raw data can be obtained from the corresponding authors upon reasonable request.

## References

[1] Patangia D.V., Anthony Ryan C., Dempsey E., Paul Ross R., Stanton C. (2022). Impact of Antibiotics on the Human Microbiome and Consequences for Host Health. MicrobiologyOpen.

[2] Baran A., Kwiatkowska A., Potocki L. (2023). Antibiotics and Bacterial Resistance—A Short Story of an Endless Arms Race. Int. J. Mol. Sci..

[3] Dashti A.A., Jadaon M.M. (2025). Extended-Spectrum Beta-Lactamases (ESBLs) Gene Mutations in Kuwait: How Much Do We Know? Not Much!. Bacteria.

[4] Chinemerem Nwobodo D., Ugwu M.C., Oliseloke Anie C., Al-Ouqaili M.T.S., Chinedu Ikem J., Victor Chigozie U., Saki M. (2022). Antibiotic Resistance: The Challenges and Some Emerging Strategies for Tackling a Global Menace. J. Clin. Lab. Anal..

[5] Uddin T.M., Chakraborty A.J., Khusro A., Zidan B.R.M., Mitra S., Emran T.B., Dhama K., Ripon M.d.K.H., Gajdács M., Sahibzada M.U.K. (2021). Antibiotic Resistance in Microbes: History, Mechanisms, Therapeutic Strategies and Future Prospects. J. Infect. Public Health.

[6] Azimi L., Armin S., Samadi Kafil H., Abdollahi N., Ghazvini K., Hasanzadeh S., Shahraki Zahedani S., Rafiei Tabatabaei S., Fallah F. (2022). Evaluation of Phenotypic and Genotypic Patterns of Aminoglycoside Resistance in the *Gram-negative bacteria* Isolates Collected from Pediatric and General Hospitals. Mol. Cell. Pediatr..

[7] Webster C.M., Shepherd M. (2022). A Mini-Review: Environmental and Metabolic Factors Affecting Aminoglycoside Efficacy. World J. Microbiol. Biotechnol..

[8] Alkofide H., Alhammad A.M., Alruwaili A., Aldemerdash A., Almangour T.A., Alsuwayegh A., Almoqbel D., Albati A., Alsaud A., Enani M. (2020). Multidrug-Resistant and Extensively Drug-Resistant Enterobacteriaceae: Prevalence, Treatments, and Outcomes—A Retrospective Cohort Study. Infect. Drug Resist..

[9] El-Far S.W., Abukhatwah M.W. (2023). Prevalence of Aminoglycoside Resistance Genes in Clinical Isolates of *Pseudomonas aeruginosa* from Taif, Saudi Arabia—An Emergence Indicative Study. Microorganisms.

[10] Boussetta A., Kharbach N., Abdellatif A., Karray A., Jellouli M., Gargah T. (2023). Predictive Factors of Urinary Tract Infections Caused by Extended-Spectrum β-Lactamase-Producing *Escherichia coli* in Children: A Prospective Tunisian Study. Tunis. Med..

[11] Derraji S., Menyeng A., Ameur A., Cherrah Y. (2015). Infections urinaires nosocomiales liées au sondage urinaire. Maroc. Méd..

[12] Merzougui L., Barhoumi T., Guizani T., Barhoumi H., Hannachi H., Turki E., Majdoub W. (2018). Les Infections Nosocomiales En Milieu de Réanimation: Incidence Annuelle et Aspects Cliniques Au Service de Réanimation Polyvalente, Kairouan, Tunisie, 2014. Pan Afr. Med. J..

[13] Maldiney T., Pineau V., Bregigeon R., Neuwirth C., Perraud F., Podac B., Blot M., Piroth L., Fournel I., Clere-Jehl R. (2024). Faire la lumière sur le biofilm en soins critiques: Un compartiment mésestimé pour lutter contre les infections associées aux dispositifs médicaux invasifs. Méd. Intensive Réanim..

[14] Muhammad A. (2020). Antibiotics Resistance of Extended Spectrum Beta Lactamases Uropathogenic *Escherichia coli* in Peshawar-Pakistan. Pure Appl. Biol..

[15] Saquib J., Alhomaidan H., Al-Mohaimeed A., Rajab A., Almazrou A., Saquib N. (2024). Gender Differences in Healthcare Status and Utilization: A Comprehensive Study on Adults in Saudi Arabia. J. Umm Al-Qura Univ. Med. Sci..

[16] Jamil J., Haroon M., Sultan A., Khan M.A., Gul N. (2018). Prevalence, Antibiotic Sensitivity and Phenotypic Screening of ESBL/MBL Producer *E. Coli* Strains Isolated from Urine; District Swabi, KP, Pakistan. J. Pak. Med. Assoc..

[17] Zhan Z.-S., Shi J., Zheng Z.-S., Zhu X.-X., Chen J., Zhou X.-Y., Zhang S.-Y. (2024). Epidemiological Insights into Seasonal, Sex-specific and Age-related Distribution of Bacterial Pathogens in Urinary Tract Infections. Exp. Ther. Med..

[18] Gu J., Chen X., Yang Z., Bai Y., Zhang X. (2022). Gender Differences in the Microbial Spectrum and Antibiotic Sensitivity of Uropathogens Isolated from Patients with Urinary Stones. Clin. Lab. Anal..

[19] Sbiti M., Lahmadi K., Louzi L. (2017). Profil Épidémiologique Des Entérobactéries Uropathogènes Productrices de Bêta-Lactamases à Spectre Élargi. Pan Afr. Med. J..

[20] Almutawif Y.A., Eid H.M.A. (2023). Prevalence and Antimicrobial Susceptibility Pattern of Bacterial Uropathogens among Adult Patients in Madinah, Saudi Arabia. BMC Infect. Dis..

[21] Whelan S., Lucey B., Finn K. (2023). Uropathogenic *Escherichia coli* (UPEC)-Associated Urinary Tract Infections: The Molecular Basis for Challenges to Effective Treatment. Microorganisms.

[22] Krapp F., García C., Hinostroza N., Astocondor L., Rondon C.R., Ingelbeen B., Alpaca-Salvador H.A., Amaro C., Aguado Ventura C., Barco-Yaipén E. (2023). Prevalence of Antimicrobial Resistance in *Gram-negative bacteria* Bloodstream Infections in Peru and Associated Outcomes: VIRAPERU Study. Am. J. Trop. Med. Hyg..

[23] Behzadi P., García-Perdomo H.A., Autrán Gómez A.M., Pinheiro M., Sarshar M. (2023). Editorial: Uropathogens, Urinary Tract Infections, the Host-Pathogen Interactions and Treatment. Front. Microbiol..

[24] Kara A., Massaro C., Giammanco G.M., Alduina R., Boussoualim N. (2024). Phylogenetic Diversity, Antibiotic Resistance, and Virulence of *Escherichia coli* Strains from Urinary Tract Infections in Algeria. Antibiotics.

[25] Goita D., Sogoba D., Ado A.I., Traore M., Fofana Y., Diarra A., Coulibaly D.S., Sangare F., Keita B.S., Cissoko Y. (2025). Profil Épidémiologique et La Résistance Aux Antibiotiques Des Bactéries Isolées Au Laboratoire d’analyses Biomédicales Du CHU Mère Enfant Le “Luxembourg” de Bamako Au Mali. Jaccr Infect..

[26] Brian J.W. Revue Générale des Bêta-Lactamines—Maladies Infectieuses. https://www.msdmanuals.com/fr/professional/maladies-infectieuses/bactéries-et-médicaments-antibactériens/revue-générale-des-bêta-lactamines.

[27] Angho J.-R.T., Fefe J.-F.B., Amegiede I.A., Iyamba J.-M.L., Takaisi-Kikuni N.B. (2024). Phenotypic Detection of Extended-Spectrum Beta-Lactamases and Antibiotic Resistance of Enterobacteriaceae Strains Isolated from Hospitalized Patients in the Ngaliema Clinic of Kinshasa, the Democratic Republic of Congo: Détection Phénotypique Des Bétalactamases à Spectre Elargi et Résistance Aux Antibiotiques Des Souches d’entérobactéries Isolées Des Malades Hospitalisés à La Clinique Ngaliema de Kinshasa, République Démocratique Du Congo. Ann. Afr. De Med..

[28] Ugwuanyi F.C., Ajayi A., Ojo D.A., Adeleye A.I., Smith S.I. (2021). Evaluation of Efflux Pump Activity and Biofilm Formation in Multidrug Resistant Clinical Isolates of *Pseudomonas aeruginosa* Isolated from a Federal Medical Center in Nigeria. Ann. Clin. Microbiol. Antimicrob..

[29] Deku J.G., Duedu K.O., Ativi E., Kpene G.E., Feglo P.K. (2022). Burden of Fluoroquinolone Resistance in Clinical Isolates of *Escherichia coli* at the Ho Teaching Hospital, Ghana. Ethiop. J. Health Sci..

[30] Hailemariam M., Alemayehu T., Tadesse B., Nigussie N., Agegnehu A., Habtemariam T., Ali M., Mitiku E., Azerefegne E. (2021). Major Bacterial Isolate and Antibiotic Resistance from Routine Clinical Samples in Southern Ethiopia. Sci. Rep..

[31] Vaez H., Faghri J., Nasr Esfahani B., Moghim S., Fazeli H., Sedighi M., Ghasemian Safaei H. (2015). Antibiotic Resistance Patterns and Genetic Diversity in Clinical Isolates of *Pseudomonas aeruginosa* Isolated from Patients of a Referral Hospital, Isfahan, Iran. Jundishapur J. Microbiol..

[32] Diop M., Bassoum O., Ndong A., Wone F., Ghogomu Tamouh A., Ndoye M., Youbong T., Mbacké Daffé S.M., Radji R.O., Gueye M.W. (2025). Prevalence of Multidrug-Resistant Bacteria in Healthcare and Community Settings in West Africa: Systematic Review and Meta-Analysis. BMC Infect. Dis..

[33] L’OMS Met à Jour la Liste des Bactéries Résistantes aux Médicaments qui Représentent la Plus Grande Menace Pour la Santé Humaine. https://www.who.int/fr/news/item/17-05-2024-who-updates-list-of-drug-resistant-bacteria-most-threatening-to-human-health.

[34] Chakuzira W., Okoche J.M.M., Mkansi M. (2024). Refining the Definition and Typologies of Entrepreneurship in Africa: A Systematic Review. Adm. Sci..

[35] Ahmed O.B., Asghar A.H., Bamaga M., Bahwerth F.S., Ibrahim M.E. (2023). Characterization of Aminoglycoside Resistance Genes in Multidrug-Resistant *Klebsiella pneumoniae* Collected from Tertiary Hospitals during the COVID-19 Pandemic. PLoS ONE.

[36] Saeli N., Jafari-Ramedani S., Ramazanzadeh R., Nazari M., Sahebkar A., Khademi F. (2024). Prevalence and Mechanisms of Aminoglycoside Resistance among Drug-Resistant *Pseudomonas aeruginosa* Clinical Isolates in Iran. BMC Infect. Dis..

[37] Altayb H.N., Elbadawi H.S., Alzahrani F.A., Baothman O., Kazmi I., Nadeem M.S., Hosawi S., Chaieb K. (2022). Co-Occurrence of β-Lactam and Aminoglycoside Resistance Determinants among Clinical and Environmental Isolates of *Klebsiella pneumoniae* and *Escherichia coli*: A Genomic Approach. Pharmaceuticals.

[38] Muteeb G. (2023). Network Meta-Analysis of Antibiotic Resistance Patterns in Gram-Negative Bacterial Infections: A Comparative Study of Carbapenems, Fluoroquinolones, and Aminoglycosides. Front. Microbiol..

[39] Castanheira M., Kimbrough J.H., Lindley J., Doyle T.B., Ewald J.M., Sader H.S. (2024). In Vitro Development of Resistance against Antipseudomonal Agents: Comparison of Novel β-Lactam/β-Lactamase Inhibitor Combinations and Other β-Lactam Agents. Antimicrob. Agents Chemother..

[40] Bodendoerfer E., Marchesi M., Imkamp F., Courvalin P., Böttger E.C., Mancini S. (2020). Co-Occurrence of Aminoglycoside and β-Lactam Resistance Mechanisms in Aminoglycoside- Non-Susceptible *Escherichia coli* Isolated in the Zurich Area, Switzerland. Int. J. Antimicrob. Agents.

[41] Livermore D.M., Day M., Cleary P., Hopkins K.L., Toleman M.A., Wareham D.W., Wiuff C., Doumith M., Woodford N. (2019). OXA-1 β-Lactamase and Non-Susceptibility to Penicillin/β-Lactamase Inhibitor Combinations among ESBL-Producing *Escherichia coli*. J. Antimicrob. Chemother..

[42] Maione A., Galdiero E., Cirillo L., Gambino E., Gallo M.A., Sasso F.P., Petrillo A., Guida M., Galdiero M. (2023). Prevalence, Resistance Patterns and Biofilm Production Ability of Bacterial Uropathogens from Cases of Community-Acquired Urinary Tract Infections in South Italy. Pathogens.

[43] Dumaru R., Baral R., Shrestha L.B. (2019). Study of Biofilm Formation and Antibiotic Resistance Pattern of Gram-Negative Bacilli among the Clinical Isolates at BPKIHS, Dharan. BMC Res. Notes.

[44] Joshi P.A., Rajmane A., Shikhare V., Ramteerthakar M., Kulkarni V. (2021). A Study of Biofilm Production and Antimicrobial Susceptibility Pattern among Urinary Isolates. Indian J. Microbiol. Res..

[45] Belbase A., Pant N.D., Nepal K., Neupane B., Baidhya R., Baidya R., Lekhak B. (2017). Antibiotic Resistance and Biofilm Production among the Strains of *Staphylococcus aureus* Isolated from Pus/Wound Swab Samples in a Tertiary Care Hospital in Nepal. Ann. Clin. Microbiol. Antimicrob..

[46] Karigoudar R.M., Karigoudar M.H., Wavare S.M., Mangalgi S.S. (2019). Detection of Biofilm among Uropathogenic *Escherichia coli* and Its Correlation with Antibiotic Resistance Pattern. J. Lab. Physicians.

[47] Macias-Valcayo A., Aguilera-Correa J.-J., Broncano A., Parron R., Auñon A., Garcia-Cañete J., Blanco A., Esteban J. (2022). Comparative In Vitro Study of Biofilm Formation and Antimicrobial Susceptibility in Gram-Negative Bacilli Isolated from Prosthetic Joint Infections. Microbiol. Spectr..

[48] Murthy N.S., Mahale R.P., Rao A. (2024). Comparative Study of *Staphylococcus aureus* Biofilm Formation among Clinical Isolates and Nasal Colonisers. J. Pure Appl. Microbiol..

[49] Ndako A.C., Nasiru A.U., Mamman G.P., Friday A., Egbede N.D., Kin A., Suleiman R., Idris A. (2020). The roles of biofilm in antibiotics resistance. Int. J. Adv. Res..

[50] Bertelloni F., Cagnoli G., Ebani V.V. (2021). Virulence and Antimicrobial Resistance in *Canine staphylococcus* Spp. Isolates. Microorganisms.

[51] Mathur A., Kay C., Xue Y., Pandey A., Lee J., Jing W., Enosi Tuipulotu D., Lo Pilato J., Feng S., Ngo C. (2023). *Clostridium perfringens* Virulence Factors Are Nonredundant Activators of the NLRP3 Inflammasome. EMBO Rep..

[52] Rahman A., Sardar S., Niaz Z., Khan A., Sheheryar S., Alrefaei A.F., Hamayun M., Ali S. (2024). Lipase and Protease Production Ability of Multi-Drug Resistant BacteriaWorsens the Outcomes of Wound Infections. Curr. Pharm. Des..

[53] Amara M., Aubin G., Caron F., Cattoir V., Dortet L., Goutelle S., Jeannot K., Lepeule R., Lina G., European Committee on Antimicrobial Susceptibility Testing (EUCAST) (2022). French Copyright Centre (FCC): Paris, France. https://www.eucast.org/.

[54] Woodman M.E., Savage C.R., Arnold W.K., Stevenson B. (2016). Direct PCR of Intact Bacteria (Colony PCR). Curr. Protoc. Microbiol..

[55] Coy M.R., Hoffmann M., Kingdom Gibbard H.N., Kuhns E.H., Pelz-Stelinski K.S., Stelinski L.L. (2014). Nested-Quantitative PCR Approach with Improved Sensitivity for the Detection of Low Titer Levels of Candidatus Liberibacter Asiaticus in the Asian Citrus Psyllid, *Diaphorina citri* Kuwayama. J. Microbiol. Methods.

[56] Kim J., Jeon S., Rhie H., Lee B., Park M., Lee H., Lee J., Kim S. (2009). Rapid Detection of Extended Spectrum β-Lactamase (ESBL) for Enterobacteriaceae by Use of a Multiplex PCR-Based Method. Infect. Chemother..

[57] Castello A., Massaro C., Seghers E., Ferraro C., Costa A., Alduina R., Cardamone C. (2025). Isolation and Molecular Characterization of Antimicrobial-Resistant Bacteria from Vegetable Foods. Pathogens.

[58] Stepanović S., Vuković D., Hola V., Bonaventura G.D., Djukić S., Ćirković I., Ruzicka F. (2007). Quantification of Biofilm in Microtiter Plates: Overview of Testing Conditions and Practical Recommendations for Assessment of Biofilm Production by Staphylococci. APMIS.

[59] Türkel İ., Yıldırım T., Yazgan B., Bilgin M., Başbulut E. (2018). Relationship between Antibiotic Resistance, Efflux Pumps, and Biofilm Formation in Extended-Spectrum β-Lactamase Producing *Klebsiella pneumoniae*. J. Chemother..

[60] Mogrovejo-Arias D.C., Brill F.H.H., Wagner D. (2020). Potentially Pathogenic Bacteria Isolated from Diverse Habitats in Spitsbergen, Svalbard. Environ. Earth Sci..

[61] Tula M., Filgona J., Kyauta S., Elisha R. (2023). Screening for Some Virulent Factors among Bacterial Isolates from Surfaces of Hospital Fomites and Hands of Healthcare Workers. Cell. Mol. Biomed. Rep..

[62] Riffel A., Brandelli A. (2006). Keratinolytic Bacteria Isolated from Feather Waste. Braz. J. Microbiol..

[63] AlDoori I.H.A., Mahal J.D., Maaroof M.N. (2020). Determination of Genes Responsible for Some Virulence Factors of Bacteria Isolated from Contaminated Groundwater. EurAsian J. Biosci..

